# *Encephalitozoon cuniculi* Infection in Rabbits (*Oryctolagus cuniculus*): Data from an International Survey of Exotic and Small Animal Veterinarians

**DOI:** 10.3390/ani14223295

**Published:** 2024-11-15

**Authors:** Fabiano Montiani-Ferreira, Anja Joachim, Frank Künzel, Franz Riegler Mello, Emma Keeble, Jennifer Graham, Jaume Martorell, Jean-Francois Quinton, Ann Gottenger, Carolyn Cray

**Affiliations:** 1 Departamento de Medicina Veterinária, Universidade Federal do Paraná, Curitiba 80035-050, PR, Brazil; 2 Division of Comparative Pathology, Department of Pathology & Laboratory Medicine, University of Miami Miller School of Medicine, Miami, FL 33136, USA; 3 Institute of Parasitology, Department of Pathobiology, University of Veterinary Medicine, 1210 Vienna, Austria; 4 Clinic for Small Animals, Clinical Department for Small Animals and Horses, University of Veterinary Medicine, 1210 Vienna, Austria; 5 The Royal (Dick) School of Veterinary Studies, The Roslin Institute, The University of Edinburgh, Midlothian EH25 9RG, UK; 6 Graham Veterinary Consulting, LLC, Madison, AL 35758, USA; 7 Fundació Hospital Clínic Veterinari, Facultat de Veterinària, Universitat Autònoma de Barcelona, 08193 Cerdanyola del Vallès, Spain; 8 Departament de Medicina i Cirurgia Animals, Facultat de Veterinària, Universitat Autònoma de Barcelona, 08193 Cerdanyola del Vallès, Spain; 9 ADVETIA Centre Hospitalier Vétérinaire, 78140 Vélizy Villacoublay, France; 10 College of Veterinary Medicine and Public Health, The Ohio State University, Columbus, OH 43210, USA

**Keywords:** *Encephalitozoon cuniculi*, epidemiology, geographical differences, health survey, lagomorph, microsporidia, questionnaire, rabbit, survey, treatment

## Abstract

In 2022–2023, the first survey of clinicians regarding *Encephalitozoon cuniculi* infection of rabbits was undertaken. The purpose was to assess the current worldwide knowledge of clinicians regarding influence of breed and age, clinical presentations, use of various diagnostic tools, and treatment regimens and to explore how responses may differ by geographic location. Identifying mostly as exotic animal clinicians, 339 respondents participated, with nearly half from the United States and the remainder from 35 other countries. Neurological signs were most consistently reported as associated with *Encephalitozoon cuniculi* and the most recurrent clinical case presentation. Other responses showed differences by geographic location. For example, age predilection for disease varied, with 71.1% of international respondents indicating that infection was more common in young rabbits versus 28.9% who suggested it was more frequent in older rabbits. This was significantly different (*p* = 0.001) from the information from respondents from the United States, where 48.0% indicated that younger rabbits had an increased incidence versus 52.0% who indicated that older age increased occurrence. While some differences may be related to the practice composition and the training level of respondents, many responses were in alignment with the existing literature. Future inquiries should focus more on these discrepancies to aid in generating guidelines for the diagnosis and treatment of this infectious disease.

## Simple Summary

For many diseases and infections, practitioners of veterinary medicine draw upon the established literature and anecdotal reports. In rabbits, infection with *Encephalitozoon cuniculi*, a microsporidian parasite, can result in severe disease. While several comprehensive reviews have been published on this infectious agent and resultant disease, there continue to be many challenges in antemortem diagnosis and treatment which has led to some variability in protocols. Using an international survey of veterinary clinicians, we found both differences and consistencies by geographic location which may reflect the influences of client/practice composition and the education/training level of respondents. The results should aid in generating guidelines and education initiatives involving this infectious disease.

## 1. Introduction

*Encephalitozoon cuniculi* is an obligate intracellular microsporidian parasite [1]. While it appears to have a wide host range in mammals, including humans, the rabbit is most known for the development of clinical disease, which can often be severe [2,3,4,5,6,7]. Several comprehensive review papers have been published on the course of infection, disease, diagnostics, and treatment in the past 15 years [2,3,4,5,6,7]. Despite this bank of information, some clinicians feel there are challenges in antemortem diagnosis and treatment and view the limited older studies and the paucity of newer studies with some degree of skepticism. Anecdotally, this has led to some variability in treatment protocols. In addition, options for diagnostic assays and advanced imaging techniques may be limited or differ in many regions of the world. A lack of practice standards and formal guidelines for evaluation, diagnosis, and treatment are also confounding factors.

The objective of this survey was to assess the knowledge base of clinicians treating rabbits with presumed or confirmed *E. cuniculi* disease with an additional goal of examining responses by the geographic location of the respondent. It was expected that comparing the experiences and perceptions of the respondents would form a unified basis of guidelines for the diagnosis and treatment of encephalitozoonosis of rabbits. In addition, information obtained from the survey may also reveal areas of research and education that are lacking and thus may provide a foundation for initiatives to improve diagnostic and treatment regimens.

## 2. Materials and Methods

### 2.1. Data Collection

An online survey was created using Microsoft Forms 2022 (Microsoft, Redmond, WA 98052, USA) and was available for completion from 15 November 2022 to 18 February 2023 (see Supplemental File). The survey was available in English, Spanish, French, German, and Portuguese. A link to the form was distributed via email and social media as well as being present on the website of the Association of Exotic Mammal Veterinarians. The first section asked respondents about the country where they practice, the number of rabbits seen per month, and if they identify as an exotic animal or small animal clinician. Questions regarding board certification or other specialization were not included. The form included a total of 27 questions in several formats: multiple choice, including dichotomous “yes” or “no” alternatives; frequency using descriptors including never, sometimes, often, and always; and numerical scales to rate knowledge or experience (from 1 to 10 or from 1 to 5 where 1 = never; 2 = rarely; 3 = sometimes; 4 = often; 5 = always), as well as open-ended questions, plus an option to provide an email address for follow-up communication. Based on some responses to the first survey regarding treatment regimen, a second survey was conducted from 28 June to 16 July 2023 (see Supplemental File). This second form included 18 questions delving further into treatments and relapsing infection, and a link was sent by email to the original respondent group.

### 2.2. Respondent Demographics

**Figure 1:**
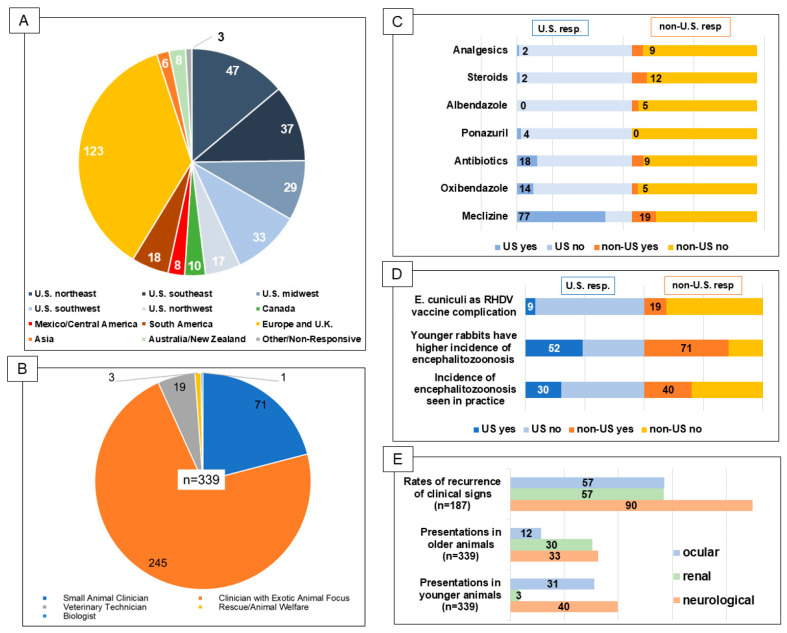
(**A**) Respondent demographics—location. U.S. respondents—48.1%; non-U.S. respondents—51.9%. (**B**) Respondent demographics—professional classification. (**C**) Treatment regimens with significant differences by respondent location (percentage of respondents in the U.S. versus non-U.S.). Analgesics, *p* = 0.007; steroids, *p* = 0.001; albendazole, *p* = 0.003; ponazuril, *p* = 0.013; antibiotics, *p* = 0.03; oxibendazole, *p* = 0.004; meclizine, *p* < 0.0001 (**D**) Queries with significant differences by respondent location (percentage of respondents in the U.S. versus non-U.S.). RHDV query, *p* < 0.0001, incidence of encephalitozoonosis, *p* = 0.02. In the younger animal case presentation query, the difference was present in the non-U.S. responders only, *p* = 0.001. (**E**) Queries with significant differences. Values represent percentages. All queries, *p* < 0.0001.

A total of 339 responses were collected for the first survey. Forty-eight percent (163/339) of respondents were from the United States (Figure 1A). Of the remaining respondents, 51.9% (176/339) represented 35 other countries. Respondents from Europe (13.9%), including Spain, the United Kingdom, and France as the top responses, represented most of the international respondents, while the remainder of the responses were from practitioners from Canada, Asia, South America, Central America, Africa, and Australia. Most respondents identified as clinicians with an exotic animal focus 72.3% (245/339). This identification was not restricted by board certification. A total of 20.9% (71/339) of the respondents identified as small-animal-focused. There was not a significant difference in the proportion of the small animal clinicians (*p* = 0.53, 34/176 versus 37/163) and exotic animal clinicians (*p* = 0.34, 137/176 versus 108/163) outside and within the United States, respectively. However, within the United States, significantly more veterinary technicians responded compared to outside the United States (14/163 versus 5/176, respectively, *p* = 0.025). While veterinary technicians represented a small portion of the overall response (5.6%—19/339), individuals in animal welfare and rehabilitation (0.8%—3/339) and one biologist (0.3%—1/339) also completed the survey (Figure 1B).

The follow-up survey received 138 responses of which 52.2% were from respondents in the United States and 83.3% of the responses were from exotic animal veterinarians.

### 2.3. Data Management and Analysis

Data were downloaded into Microsoft Excel Version 2311 (Microsoft, Redmond, WA 98052, USA). The normality of the distribution of continuous numerical data was evaluated using the D’Agostino–Pearson Test. This test was chosen to assess skewness and kurtosis, providing a comprehensive evaluation of data distribution. For non-normally distributed numerical data, such as number of rabbits seen per month or percentage of rabbit patients suspected to have encephalitozoonosis and results of the numerical scales, Mann–Whitney U tests were used. The Mann–Whitney U test was selected to compare medians between two independent groups when the data did not meet normal distribution assumptions. In addition, for non-normally distributed data, the median and interquartile range (IQR) was used to express results. The numerical scale type of data was further analyzed in two ways, such as the previously used Likert scale [8]. First, nonparametric procedures based on the scale were applied to maintain the validity of analysis without relying on distributional assumptions and second, by comparing proportions of results, with options varying from 0 (never) to 5 (frequently), by selecting scores 3 and/or above. These were considered the upper strata and were compared to the answers of the lower strata (scores 2 and below). These variables were then organized into contingency tables and were compared using the chi-square tests or Fisher’s exact test. Chi-square tests were chosen for larger sample sizes (when the expected cell counts are all at least 10) to evaluate the independence of categorical variables, while Fisher’s exact test was applied for smaller sample sizes, to ensure accurate *p*-value calculations, since the latter is more accurate than the chi-square test when the expected cell frequencies are small. The comparison of the responses obtained from veterinarians by geographic location were also performed using chi-square or Fisher’s exact test. A *p*-value of less than a predetermined alpha was considered a statistically significant result. Data were analyzed using MedCalc software (MedCalc Software Ltd., Ostend, Belgium; version 22.014).

## 3. Results

### 3.1. Questions Regarding Rabbits and E. cuniculi Infection

The median number of rabbits seen per month by U.S. respondents and non-U.S. respondents was identical but presented a great variation, 30 (IQR 35) and 30 (IQR 48.5), respectively. When queried on the percentage of rabbit patients suspected to have encephalitozoonosis, the median response from the U.S. was 30% (IQR 30% of the rabbit patients) and the median response from outside the U.S. was 40% (IQR 40% of the rabbit patients). This finding was significantly different, *p* = 0.02 (Figure 1D).

Fifteen options were provided for various rabbit breeds and crossbreeds, and the respondents were asked to rank the breeds that were most seen as patients. Both in the U.S. and outside the U.S., the top three responses were lop (U.S. 95.7% (156/163); non-U.S. 86.9% (153/176)), dwarf (U.S. 88.3% (144/163); non-U.S. 84.1% (148/176)), and crossbred (85.9% (140/163), non-U.S. 87.5% (154/176)). Fourteen percent (48/339) of respondents stated there was a breed predilection for infection. Clinicians from the U.S. stated that three breeds were most common in regards to ECUN infection: lop 96.3% (26/27), crossbreed 92.6% (25/27), and dwarf 85.2% (23/27). Outside the U.S., respondents stated that crossbreed 80.9% (17/21), lop 80.9% (17/21), and dwarf 76.2% (16/21) rabbit breeds as the most common.

The top three diagnoses associated with head tilt, with no difference between U.S. and non-U.S. respondents; responses were as follows: *E. cuniculi* infection (U.S. 87.1% (142/163); non-U.S., 92.1% (162/176)), otitis media/interna (U.S. 59.5% (97/163); non-U.S. 81.8% (144/176)), and concurrent otitis and *E. cuniculi* infection (U.S. 59.5% (97/163); non-U.S. 59.7% (105/176)). The respondents were also questioned about other causes of head tilt and those from the U.S. cited tumors (2.1%, 2/94), other parasites (12.8%, 12/94), and other infections (1.1%, 1/94). Non-U.S. respondents indicated tumors (3.3%, 4/122), other parasites (4.1%, 5/122), and encephalitis/meningitis (2.5%, 3/122). The response regarding parasites was significantly different (*p* = 0.023) in U.S. responses versus non-U.S. responses.

Responses differed by geographic location regarding clinical signs associated with *E. cuniculi* disease. For this portion of the survey, respondents were given the following options: neurological signs, renal signs, ocular signs, GI stasis, neurological/renal signs, neurological/ocular signs, and renal/ocular signs. Within the U.S., clinicians reported clinical signs, in order from most to least common: neurological, ocular, gastrointestinal, and combined neurological/ocular. Outside the U.S., the ranking was as follows: neurological, ocular, gastrointestinal, and renal. Regardless of geographic location, approximately half of the respondents, U.S. 54.0% (88/163) and non-U.S. 51.1% (90/176), stated that GI stasis could be observed with *E. cuniculi* infection in the absence of other clinical signs normally associated with infection (i.e., neurological, ocular, renal). The most common ocular presentation was a combination of uveitis and cataracts in 88.2% (75/85) of cases. This was followed by uveitis alone 31.8% (27/85) and cataracts alone 22.4% (19/85).

When asked about age predilection for encephalitozoonosis, 46.0% (75/163) of respondents from the U.S. and 47.2% (83/176) of respondents from outside the U.S. answered that there was an age predilection. These proportions were not significantly different (*p* = 0.89). When examining the responses from the U.S. group, no significant difference (*p* = 0.70) was noted, with 48.0% (36/75) indicating that younger rabbits have an increased incidence of clinical disease versus 52.0% (39/75) indicating that an older age resulted in increased occurrence. However, when examining the responses from outside the U.S., a significantly greater number of respondents (*p* = 0.001) indicated a higher incidence in younger rabbits (71.1% (59/83)) versus in older rabbits (28.9% (24/83)) (Figure 1D).

Respondents were queried regarding the most common signs by age group (Figure 1E). Neurological and ocular signs were the most common case presentations in younger rabbits. In this group, neurological signs were reported by 36.8% (60/163) of respondents in the U.S. and 42.6% (75/176) outside the U.S. Ocular signs were reported as 25.8% (42/163) in the U.S. and 36.4% (64/176) outside the U.S. The presence of renal disease/signs in the U.S. were reported in 4.9% (8/163) of cases, while outside the U.S., it was 1.7% (3/176). Overall, neurological signs (U.S. and non-U.S.) were significantly more common compared to the observation/diagnosis of renal disease (135/339 versus 11/339, *p* < 0.0001) in younger rabbits.

In older animals, neurological signs were reported in 38.0%, (62/163) in the U.S. and 27.8% (49/176) outside the U.S. The presence of renal disease was reported in 22.1% (36/163) of the cases in the U.S. and 38.0% (67/176) in non-U.S. rabbits. Ocular signs in older animals were reported in 13.5% (22/163) of the U.S. cases and in 9.7% (17/176) of non-U.S. cases. Overall, neurological signs (U.S. and non-U.S. cases) were observed in 32.7% (111/339) versus 11.5% (39/339) of cases with ocular signs; this was a significant difference (*p* < 0.0001). Renal disease in this age group was observed in 30.3% (103/339) of cases, which was significantly (*p* < 0.0001) more common than ocular signs but not significantly more common than neurological signs (*p* = 0.6) (Figure 1E).

### 3.2. Questions Regarding Treatment for E. cuniculi Infection

Respondents were queried concerning the top four medications used for treatment. In order from most to least, U.S. respondents indicated the following: fluids (98.2%, 160/163), meloxicam (96.3%, 157/163), fenbendazole (93.3%, 152/163), and meclizine (76.7%, 125/163). Outside the U.S., the responses were fenbendazole (96.6%, 170/176), fluids (92.6%, 163/176), meloxicam (88.6%, 156/176), and maropitant (68.2%, 120/176). Within the U.S., compared to outside the U.S., meclizine (125/163 versus 33/176, *p* < 0.0001), oxibendazole (23/163 versus 8/176, *p* = 0.004), antibiotics (29/163 versus 16/176, *p* = 0.03), and ponazuril (6/163 versus 0/176, *p* = 0.013) were more commonly prescribed. Outside the U.S., albendazole (9/176 versus 0/163, *p* = 0.003), steroids (21/176 versus 4/163, *p* = 0.001), and analgesics (15/176 versus 3/163, *p* = 0.007) were more commonly used than within the U.S. (Figure 1C). Respondents identifying as exotic animal clinicians (115/339) and as small animal clinicians (18/339) used the following supportive care drugs, respectively: meloxicam, 92.2% (106/115) and 88.9% (16/18), *p* = 0.6; midazolam, 60.9% (70/115) and 44.4% (8/18), *p* = 0.3; meclizine, 62.6% (72/115) and 33.3% (6/18), *p* = 0.1; and oxibendazole, 7.8% (9/115) and 22.2% (4/18), *p* = 0.1. Considering the overall use by all clinicians, a significant difference (*p* = 0.01) was present with the more common use of meloxicam (122/133) over midazolam (78/133).

Respondents had the option of selecting treatments: fenbendazole; meloxicam; oxibendazole; fluids; meclizine; maropitant; midazolam; other benzimidazoles; other treatment. The most common treatment regimens were fenbendazole (93.3%, 152/163, for U.S. respondents; 96.6%, 170/176, for non-U.S. respondents) or a combination of fenbendazole and meloxicam (meloxicam: 96.3%, 157/163, for U.S. respondents; 88.6%, 156/176, for non-U.S. respondents). This combination was frequently used but did not show a statistically significant improvement in outcomes compared to fenbendazole alone, as indicated by respondents, where more than 70% of rabbits were considered successfully treated (*p* = 0.37). Furthermore, the use of fenbendazole alone was also not associated with significantly better outcomes (*p* = 0.90). Notably, there was an extremely small number of respondents, indicating that fenbendazole was rarely or never used (*n* = 4). Nevertheless, this group reported a similar median recurrence rate of 45% (IQR 75%), as compared to 50% reported by the predominant fenbendazole using respondents (IQR 59.5%, *p* = 0.89).

Bone marrow complications with the use of fenbendazole were reported as being rarely observed by 33.1% (71/163) of U.S. respondents and 49.4% (65/176) of non-U.S. respondents. Overall, the majority 58% (198/339) of respondents reported that these complications were never observed. A recheck of patients via hematology was frequently completed by 39.5% (134/339) of respondents. In comparison, 45.7% (155/339) stated that rechecks were rarely performed, and 14.7% (50/339) reported that rechecks were not performed at all.

### 3.3. Other Questions

Respondents from the U.S. stated that the recurrence of infection occurs frequently, namely 60.7% (99/163) of the cases versus 50.0% (88/176) of cases suggested by non-U.S. respondents. Neurological presentation was significantly the most common type of clinical sign during recurrence (89.8%, 168/187, *p* < 0.0001) followed by renal signs (56.7%, 106/187) and ocular signs (57.2%, 107/187) (Figure 1E). Regarding GI stasis cases (in the absence of more common *E. cuniculi* clinical signs), 40.5% (32/79) of respondents indicated recurrence in less than one third of cases versus 44.3% (35/79) of respondents stating observed recurrence in one to two thirds of cases and 25.2% (12/79) of respondents stating that recurrence occurred in more than two thirds of cases.

The most common testing options for infection were ranked as follows: physical examination, serology, imaging, polymerase chain reaction assays, and necropsy. The use of physical examination was reported by 96.3% (157/163) of U.S. respondents and 90.3% (159/176) of non-U.S. respondents. Serological testing was reported by 82.2% (134/163) of U.S. respondents and 73.9% (130/176) of non-U.S. respondents. The use of imaging was reported by 63.2% (103/163) of U.S. respondents and 57.4% (101/176) of non-U.S. respondents. PCR was reported by 34.9% (57/163) of U.S. respondents and 26.1% (46/176) of non-U.S. respondents. Necropsy was reported by 33.1% (54/163) of U.S. respondents and 21.6% (38/176) of non-U.S. respondents. The use of these tests did not show a statistically significant difference by geographic location.

The respondents were asked to identify on a scale of 0 (0%) to 10 (100%) whether the onset of clinical signs was linked to a particular stressor. A total of 56% (92/163) of U.S. respondents and 44.3% (78/176) of non-U.S. respondents stated that such a link was seen in less than 30% of cases. Similarly, 36.2% (59/163) of U.S and 45.5% (80/176) of non-U.S. respondents stated that a stressor could be identified in 40–70% of cases. In contrast, 7.4% (12/163) of U.S and 10.2% (18/176) of non-U.S. respondents cited a stressor in 80–100% of cases.

The respondents were asked if they thought whether the onset of *E. cuniculi* disease was associated with vaccination. Significantly more respondents from outside the U.S. answered “yes” or “possibly” to such cases after vaccination to rabbit hemorrhagic disease virus (RHDV) when compared to respondents from the U.S. (*p* < 0.0001, Figure 1D). Non-U.S. respondents reported onset of *E. cuniculi* disease after vaccination represented 18.8% (16/138) whereas 8.5% (11/129) respondents from the U.S. reported the observation.

In response to a question regarding the possibility of zoonotic infection with *E. cuniculi*, 6.1% (10/163) of U.S. respondents and 7.4% (13/176) of non-U.S. respondents indicated that they were aware of this potential risk.

## 4. Discussion

Surveys have been implemented and reported throughout veterinary literature to examine a wide variety of areas from education and career initiatives to medication use to pet ownership. In regard to rabbits, surveys have been utilized to obtain information ranging from owner knowledge of rabbit husbandry and health [9], causes of rabbit mortality [10,11], and veterinary confidence in treating exotic pets [12]. The present report represents the first survey on *E. cuniculi* infection.

The responses of the current survey were examined by demographics, including location, namely grouped by U.S. and non-U.S., as well as the identification of respondents as exotic animal or small animal clinicians. While the number of U.S. and non-U.S. responses was nearly the same, it is acknowledged that respondents from Europe dominated the non-U.S. group. The number of respondents from these areas was likely related to the call for response to the survey, which was distributed through emails to available databases, social media, and publicizing by exotic animal veterinary associations. In addition, the density of pet rabbits and available veterinary care in these areas is also likely higher [13,14,15]. Furthermore, the survey was also dominated by respondents who identified as clinicians with an exotic animal focus. While this did not exclude those who were not board-certified in this specialty area, the higher number of responses is also likely linked to locations where caseloads with a higher number of exotic pets would be expected and where further education and clinical experience for these species could be obtained. To this point, the overall median number of rabbits with encephalitozoonosis seen per month by respondents was approximately 30% but, interestingly, there was a significant difference by location, with a higher percentage reported by non-U.S. respondents. This may be related to clinician education and experience level as well as different levels of infection by country (seroprevalence reports range from 31.6 to 87.1%) and possible strains of *E. cuniculi* with different pathogenicities [16].

Respondents were asked about top diagnoses associated with head tilt and the results were as follows: encephalitozoonosis, otitis media/interna, and a combined etiology. This response is consistent with the knowledge that otitis media/interna is a primary differential diagnosis in rabbits that present with vestibular signs [2,3,17]. Other diagnoses that were cited by respondents included other infections, trauma, neoplasia, other parasites, and meningoencephalitis. These findings are consistent with that reported in a retrospective pathology study of rabbits with neurological disease [18].

The top three breeds of rabbits that were seen within and outside the U.S. were the same but were ranked differently. Outside the U.S., the crossbred rabbit ranked highest followed by the lop and dwarf breeds. This is consistent with a survey of rabbit veterinary care in England [11] but different to the data reported in a study of pet rabbit mortality in Japan, which indicated dwarf, lop, and mini rex as the top three breeds [10]. In contrast, U.S. respondents listed the top three breeds as lop, dwarf, and crossbred. These responses likely reflect how common these breeds are kept as pets but may be influenced by the breeds that are presented more often for veterinary care. It should be noted, however, that dwarf rabbits may not be uniformly defined by clinicians or owners as a specific breed and may include mixed-breed rabbits [19]. In the survey, respondents were given options of both dwarf and dwarf crossbred to aid in addressing this possible misidentification.

In a related question, respondents were asked if they believed there was a breed predilection for *E. cuniculi* infection. The overall response was low, and the ranking included those breeds that were most commonly seen as patients: lop, dwarf, and crossbred. Interestingly, responses are consistent with the anecdotal belief that dwarf rabbits may have a predilection for *E. cuniculi* infection and disease. This belief may stem from reports on rabbits presenting with head tilt where a study included a large seropositive group with clinical signs and histological changes which was composed solely of dwarf rabbits [17] and multiple reports focused on ocular diseases in this breed [20]. In a study from the UK, breed predilection, based on the presence of antibodies, was not observed [21]. The survey results as well as the literature may show a bias regarding the aforementioned variability in the identification of a true dwarf breed versus small-sized crossbred rabbits. In total, there is no evidence for a breed predilection in rabbits with *E. cuniculi*; the survey findings should be examined in animal models to better address this question.

Approximately one half of respondents indicated that there was an age predilection for *E. cuniculi*. Interestingly, 52% of U.S. respondents stated that older animals have an increased incidence of infection, whereas 71% of non-U.S. respondents indicated that encephalitozoonosis occurrence was higher in younger animals. This difference was statistically significant. Age was previously not found to be associated with seroprevalence in rabbits in the UK [21] although studies of very young pet rabbits and farmed rabbits did indicate age differences in Italy [22,23]. In a large study of rabbits in Germany with suspected and proven *E. cuniculi* infection as well as other diseases, age was found to be a significant difference with an increased incidence of clinical signs in older rabbits [24]. It is worth noting that the survey results may have been biased by the relative number of younger versus older animals which were seen as patients in these two locations; this information was not obtained during the survey process. In addition, the age range associated with the classification of young and old rabbits was not predefined for the respondents.

Respondents were asked about the most common clinical presentations associated with encephalitozoonosis; it should be noted that these signs are common in rabbits as a result of several disease processes. The top three presentations in order from highest to lowest were neurological, ocular, and gastrointestinal. Neurological signs have been reported to be the most common presentation and ocular signs are frequently observed in younger animals [17,25]. Gastrointestinal signs as a presentation associated with *E. cuniculi* infections have not been well represented in the literature, although in a recent report of a large number of rabbits, gastrointestinal disorders were reported in 27.5% of seropositive rabbits [26]. However, the cause of rabbit gastrointestinal syndrome has been recognized as multifactorial, and gastrointestinal stasis can be a common presentation in any clinically abnormal rabbit [27]. An acknowledged limitation of this survey is that GI stasis is a broad diagnosis in clinical practice so responses may be influenced by the lack of a proper definition of this clinical sign.

A related question was presented to uncover what type of clinical presentations are more common based on the animal’s age. Respondents indicated that younger rabbits have a significantly higher incidence of neurological and ocular presentations over renal signs and older rabbits have a significantly higher incidence of neurological and renal signs rather than ocular signs. These results agree with the literature. As described above, the most frequent presentation is neurological disease [17,25]. Ocular disease has been previously reported to occur in younger rabbits that are infected in utero [20,25]. Respondents indicated that combined uveitis and cataracts were the most common ocular presentation, which is consistent with previous reports [28,29]. *Encephalitozoon cuniculi* infection in rabbits with renal disease has been reported to be subclinical and most often identified only by necropsy or by the presence of kidney mineralization on radiographs. In the case of clinical manifestations, a positive serological result in the presence of weight loss, polyuria, and azotemia supports *E. cuniculi* as, at minimum, a cofactor [25,30]. Both neurological and renal etiologies have been reported more often in older animals [25,31]. Interestingly, U.S. respondents listed a combined neurological/ocular presentation as the fourth most common clinical presentation, whereas non-U.S. respondents listed renal presentation as most common. This may be reflective of the relative number of younger versus older animals which are seen as patients and geographical variation in longevity.

Respondents indicated that primary treatment options include fenbendazole, fluids, and meloxicam. Other medications were rated as frequently used but rankings differed between U.S. and non-U.S. respondents. Some differences may be a result of the availability of such medication in particular countries. An example is meclizine which was reported to be more commonly used by U.S. respondents. Meclizine is not available in the United Kingdom but can be obtained in some European countries. Other differences were also found—oxibendazole, antibiotics, and ponazuril were rated as more frequently prescribed in the U.S. versus albendazole, steroids, and analgesics outside the U.S. These changes may reflect a difference in education, anecdotal information, and treatment approaches. Different benzimidazoles have been described for use in rabbits with vestibular disease due to *E. cuniculi* infection, sometimes with variable efficacy [5,32]. Steroid treatment has been both supported and not supported as a treatment regimen in the literature involving both experimental and naturally infected rabbits [5]. However, glucocorticoids are no longer recommended in rabbits with neurological signs due to *E. cuniculi* infection (3,30). While many veterinarians no longer use steroids as a primary treatment for *E. cuniculi* infection, it is important to note that the survey did not query the use of an immunosuppressive versus anti-inflammatory doses in affected rabbits. When respondents were questioned regarding their use of benzimidazoles, responses included availability, positive treatment experiences, and belief in anecdotal information suggesting resistance of *E. cuniculi* to particular regimens. Antibiotics may often be prescribed if advanced diagnostics are not undertaken to differentiate *E. cuniculi* infection from bacterial (mainly otitis media/interna) infection or in cases of concurrent bacterial and *E. cuniculi* infection [7,31]. A possible reason for higher ranking of antibiotic use in the U.S. is due to an organized effort to decrease antibiotic usage in veterinary and human medicine in Europe which has been ongoing for an extended period of time [33,34]. Antibiotics are no longer recommended, especially in cases when otitis media/interna has been ruled a cause of present clinical signs (3). The use of oxibendazole by respondents self-described as small animal clinicians may be related to the availability of this drug in their practice, given its use in cats and dogs [35]. The more frequent use of meloxicam over midazolam is unsurprising as all clinical manifestations result from inflammation and hence the use of anti-inflammatory medicine, such as meloxicam, could be justified in most cases. The clinical effectiveness of meloxicam is still to be proven, however, with some authors suggesting that tissue damage is irreversible [3]. It should be noted that meloxicam is contraindicated in cases with hypotension, hypovolemia, and dehydration or with pre-existing gastrointestinal or renal disease [36]. Midazolam is primarily indicated in those rabbits with acute neurological signs associated with vestibular disease and, therefore, are usually only prescribed in animals with neurological presentation rather than ocular or gastrointestinal disease [3]. 

Respondents were given the opportunity to outline additional medications that they use in treatment. While there were no consistent responses, these included vitamin B, metoclopramide, prochlorperazine, other antihistamines, and probiotics. Importantly, vestibular disorders associated with encephalitozoonosis do not commonly reduce appetite in rabbits even if they cannot maintain an upright position. Therefore, in contrast to dogs and cats, the prevention of nausea secondary to vestibular disorders with the use of prokinetics, antiemetics, or drugs developed to control dizziness (metoclopramide, prochlorperazine, meclizine) is usually not recommended [27]. The use of ponazuril is also driven by anecdotal information. Ponazuril (a metabolite of toltrazuril) is effective in the treatment of *Sarcocystis neurona* as well as coccidial infections in rabbits and other mammals [37,38]. Notably, toltrazuril was not found to be effective in the inhibition of *E. cuniculi* using a rabbit kidney cell testing method [39]. As is frequently commented on by clinicians regarding the limited and older literature on *E. cuniculi* treatment regimens, this is an area that would benefit from thorough, updated in vitro and in vivo investigations as well as clinical practice guidelines.

A query related to treatment regimens was linked to a question on the recurrence of infection. Recurrence was reported as ‘frequent’ by 50% of U.S. respondents and 61% of non-U.S. respondents with neurological presentation being the most common clinical sign. While recurrence of clinical signs is acknowledged in the literature as a possible chronic infection [7,32], most reports on treatment efficacy focus on short term recovery data [25,40]. In the present survey, while fenbendazole and meloxicam composed the most common treatment regimen there was no significant difference in the frequency of disease recurrence with its use versus the use of fenbendazole alone. Given the move by some clinicians away from the use of fenbendazole entirely or in part, the survey responses were examined for the frequency of recurrence based on this type of treatment regimen restriction. Unfortunately, the number of non-users of fenbendazole who participated in the survey was very low, which limited the inferential analysis and conclusions. This treatment inquiry thus remains an open question that may be best examined with animal models.

Benzimidazole toxicity was previously reported in rabbits [41]. Bone marrow complications were rated as rarely observed by respondents and most responded that this adverse effect was never observed. These responses may be linked to a second query about the frequency of hematology rechecks after the start of treatment: 45.7% of respondents reported rare rechecks and 14.7% reported no rechecks.

Several other questions were included in the survey regarding diagnostic techniques. U.S. respondents commonly reported the use of serology, and this result, in part, is likely linked to the greater availability of this test through many laboratories. It should be noted that a positive serology result does not always indicate the presence of infection and thus, depending on the use and interpretation of these results, the clinician’s perception of this infection and disease may vary. The infrequent use of necropsy may be related to the lack of its election by owners and because this invasive labor-intensive process rarely confirms diagnosis. Other tools such as PCR and imaging are likely rated lower based on availability, financial constraints of owners, and regarded specificity/sensitivity [7]. Regarding the latter point, *E. cuniculi* infection cannot be definitively diagnosed through imaging, although it can be helpful to rule out other conditions in animals presenting with vestibular syndrome. Additionally, whereas PCR of the cerebral spinal fluid and urine has been shown to be unreliable as a diagnostic tool, the testing of liquified lens material is an appropriate diagnostic tool for the diagnosis of phacoclastic uveitis [25,28]. In total, these responses reflect the challenges in achieving a definitive antemortem diagnosis and could support a call for the standardization of some of these diagnostic techniques.

Stress as an inciting event of the acute onset of clinical signs was considered a major factor by most respondents, and this is consistent with a frequent hypothesis regarding *E. cuniculi* infection where the immune response is thought to regulate or suppress infection, allowing for asymptomatic chronic infections [7]. Furthermore, immune deficiency in experimental models using dexamethasone also can result in worsened infection and the onset of clinical signs [42]. In this fashion, stress may result in a downregulation of this immune response. In a related question, respondents were asked about the observed onset of *E. cuniculi* infection after vaccination for rabbit hemorrhagic disease virus (RHDV). Respondents from outside the U.S. reported a significantly higher experience with this issue versus U.S. respondents. While RHDV has been part of a vaccination protocol outside the U.S. for many years, vaccination in the U.S. has started comparatively more recently due to a RHDV2 outbreak and uses a novel emergency use authorization vaccine [43].

Lastly, respondents were asked about the possibility of zoonotic infection by *E. cuniculi*. This question did not specifically inquire about personal experience or observed zoonoses, but some respondents did relate such information. Overall, the response rating was low and more likely reflected a paucity of information in the literature and education regarding this possible issue in rabbits. Importantly, while infections may be rare in humans and are primarily reported in immune-suppressed individuals, *E. cuniculi* is a recognized zoonotic agent [44].

Surveys of this nature are flawed as they are dependent on the diversity and level of response as well as a bias towards respondents that have an interest in this subject area. Furthermore, responses in the first survey necessitated a second survey which, although distributed to the same group, did not obtain the same level of response. Considering the presumably high number of pet rabbits, the overall number of responses could be considered low so these results may not apply to all veterinarians who treat rabbits [13,45]. In this regard, the survey was dominated by exotic animal clinicians who may treat higher numbers of rabbits and have more education and access to diagnostic and treatment information for *E. cuniculi*. In addition, responses likely reflected the impressions and personal experiences of the respondents rather than precise review of medical records. These perceptions are further compounded by the challenge in definitively diagnosing this infection. Thus, the results were somewhat exposed to response and non-response biases as well as confirmation biases and this should be considered a limitation of this study [46].

## 5. Conclusions

Overall, responses to the surveys only partially reflected findings presented in the literature. It is also notable that some responses revealed an interpretation of the available literature as well as the use of anecdotal information and differences were apparent in accordance with the geographical location of the respondent. These results provide further support for education initiatives involving *E. cuniculi* infection of rabbits. Moreover, given the noted variability in treatment regimens, it is likely that the practice of veterinary medicine relative to this infectious disease would be improved through the generation of clinical practice guidelines.

## Data Availability

The raw data will be made available by the authors upon request.

## Supplementary Materials

The following supporting information can be downloaded at: https://www.mdpi.com/article/10.3390/ani14223295/s1. ECUN Surveys 1 and 2.
