# Supplementary material for: Encephalitozoon cuniculi Infection in Rabbits (Oryctolagus cuniculus): Data from an International Survey of Exotic and Small Animal Veterinarians
Source: Animals (Basel). 2024 Nov 15;14(22):3295. doi: 10.3390/ani14223295 (PMC11591500; doi:10.3390/ani14223295)
Supplement: Supplementary file 1 [file animals-14-03295-s001.zip › animals-3294213-supplementary.pdf]

E. cuniculi & Rabbits Survey

Thank you for participating in this survey. There are multiple goals of this survey:

- To query information for a consensus statement on clinical *E. cuniculi* infection.
- To build a special interest group looking to learn more about *E. cuniculi*.
- To share experiences that may aid in a larger understanding of *E. cuniculi* infection and treatment.

At the end of this survey, you may elect to provide contact information that will allow us to contact you in the future. This may include receipt of bulletins regarding research and publications about *E. cuniculi* and further detailed surveys. If you want to opt out of receiving these types of communications, you can at any time.

Note that this survey is best completed on a desktop (not phone). If you have questions or comments regarding this survey, please contact Dr. Carolyn Cray at ccray@miami.edu

* Required

1. What best describes you? *

- ◯ Veterinarian - with focus on exotic species (boarded or non-boarded)
- ◯ Veterinarian - small animal clinician
- ◯ Veterinary technician
- ◯ Rescue/animal welfare
- ◯ Biologist

2. Where do you practice in the United States? *

- ◯ Northeast
- ◯ Southeast
- ◯ Midwest
- ◯ Southwest
- ◯ Northwest
- ◯ Outside the U.S.

3. If outside the U.S.A., indicate what country

4. On average, how many rabbits do you see each month (enter a whole number)? *

5. Please rank the breeds of rabbits that you see on a 1 to 5 scale. *

	1 - Never	2	3
Lop	○	○	○
Dwarf	○	○	○
Flemish Giant	○	○	○
Rex	○	○	○
Lionhead	○	○	○
Dutch	○	○	○
Dwarf Rex	○	○	○
Dwarf Lionhead	○	○	○
Crossbred	○	○	○
New Zealand White	○	○	○
Angora	○	○	○
Dwarf Lop	○	○	○
Giant Lop	○	○	○
Dwarf Crossbred	○	○	○
Dwarf Angora	○	○	○

6. What percentage do you suspect have E. cuniculi infection? *

0	1	2	3	4	5	6	7	8	9	10

0% 100%

7. For those that present with head tilt, on a scale of 1 to 5, how common are the following diagnoses: *

	1 - Never	2	3
E. cuniculi	◯	◯	◯
Otitis media / interna	◯	◯	◯
Otitis and E. cuniculi	◯	◯	◯
Trauma	◯	◯	◯
Other	◯	◯	◯

8. If you chose other, please briefly describe the other diagnoses.

9. For those with suspected E. cuniculi, how often do you see the following clinical signs on a scale of 1 to 5 ? *

	1 - Never	2	3
Neurological signs	◯	◯	◯
Renal signs	◯	◯	◯
Ocular signs	◯	◯	◯
GI stasis	◯	◯	◯
Seizures	◯	◯	◯
Neuro + Renal	◯	◯	◯
Neuro + Ocular	◯	◯	◯
Renal + Ocular	◯	◯	◯

10. Have you seen GI stasis in the absence of clinical signs with suspected E. cuniculi cases? *

◯ Yes

◯ No

11. Do you feel that there is a breed predilection to symptomatic E. cuniculi infection? *

- ◯ No, there is no increased sensitivity
- ◯ Yes

12. If yes, please indicate which breeds.

- ☐ Lop
- ☐ Dwarf
- ☐ Flemish Giant
- ☐ Rex
- ☐ Lionhead
- ☐ Dutch
- ☐ Angora
- ☐ New Zealand White
- ☐ Crossbred
- ☐ Dwarf Crossbred

13. Do you feel like there is an age predilection to symptomatic E. cuniculi infection? *

- ◯ No
- ◯ Yes, younger rabbits
- ◯ Yes, older rabbits

14. Do you see a predilection of case presentations in younger animals? *

- ◯ More ocular cases
- ◯ More renal cases
- ◯ More neurological cases
- ◯ No

15. Do you see a predilection of case presentations in older animals? *

- ◯ More ocular cases
- ◯ More renal cases
- ◯ More neurological cases
- ◯ No

16. In what percentage of cases were you able to identify a stressor that may have resulted in the onset of clinical signs? *

0	1	2	3	4	5	6	7	8	9	10

0% 100%

17. How often do you use the following treatments in E. cuniculi suspect rabbits? *

	Almost Always	Sometimes
Panacur fenbendazole	◯	◯
Meloxicam	◯	◯
Oxibendazole	◯	◯
Fluids	◯	◯
Meclizine	◯	◯
Maropitant	◯	◯
Midazolam	◯	◯
Other	◯	◯
Other benzimidazoles	◯	◯

18. If you chose other, please briefly describe these medications

19. When using fenbendazole as a primary medication, how often have you observed bone marrow complications? *

- ◯ Never
- ◯ Rarely
- ◯ Frequently

20. When use fenbendazole as a primary medication, in how many cases do you recheck hematology after starting treatment? *

- ◯ Never
- ◯ Rarely
- ◯ Frequently

21. How often do you encounter relapsing cases on a scale of 1 to 5? *

	1-Never	2	3
Renal	◯	◯	◯
Neurological	◯	◯	◯
Ocular	◯	◯	◯
Overall Cases	◯	◯	◯

22. On a scale of 1 to 5, how often do you use these options in E. cuniculi cases? *

	1-Never	2	3
Physical Exam & History	◯	◯	◯
Serology	◯	◯	◯
PCR	◯	◯	◯
Imaging	◯	◯	◯
Necropsy	◯	◯	◯

23. If you have been using the RHDV vaccine, have you seen any cases with an onset of *E. cuniculi* following vaccination? *

- ◯ Yes
- ◯ No
- ◯ Maybe
- ◯ Does not apply to my practice

24. Have you seen any cases with an onset of *E. cuniculi* following these vaccinations? Check all that apply. *

- ☐ Myxomatosis vaccine
- ☐ Myxomatotis + RHDV vaccine
- ☐ Does not apply to my practice

25. Are you aware of any suspected or confirmed zoonotic E. cuniculi cases? *

- ◯ Yes
- ◯ No
- ◯ Maybe

26. Feel free to add any other comments here.

27. Please provide your email if you would be willing to participate in additional surveys and receive information regarding this survey and other *E. cuniculi* information.

This content is neither created nor endorsed by Microsoft. The data you submit will be sent to the form owner.

Microsoft Forms

E. cuniculi and Rabbits Survey - Part 2

Thanks for your contribution to our first survey. We will present our results at this year's ExoticsCon meeting and are drafting a paper that we will share with everyone. The first survey prompted a few more new questions as well as the need to revisit a couple of the previous questions. We thank you for your time.

Questions/comments - Dr. Carolyn Cray ccray@miami.edu

* Required

1. Where do you practice? *

- ◯ United States
- ◯ Outside the United States

2. If outside the United States, indicate what country.

3. What best describes you? *

- ◯ Veterinarian with exotic species focus (boarded or non-boarded)
- ◯ Veterinarian - small animal clinician
- ◯ Veterinary Technician
- ◯ Rescue/animal welfare
- ◯ Biologist

4. On average, how many rabbits do you see each month? *

5. How often do you use the following treatments in E. cuniculi suspect rabbits? *

	Always	Sometimes
Panacur/ fenbendazole	○	○
Meloxicam	○	○
Oxibendazole	○	○
Fluids	○	○
Meclizine or other antihistamine	○	○
Maropitant	○	○
Midazolam	○	○
Steroids	○	○
Antibiotics	○	○
Analgesics	○	○
Antiprotozoal meds	○	○
other	○	○

6. If you chose other, please describe these medications.

7. If you chose oxibendazole. please describe if you use this over fenbendazole and why.

8. What percentage of E. cuniculi patients do you feel you have successful (non relapsing) treatments (0=0%, 10=100%)? *

0	1	2	3	4	5	6	7	8	9	10

0% 100%

9. When thinking more about question 8, how often do you have successful treatments (non-relapsing) by clinical signs? *

	1-Never	2	3
Neurological signs	○	○	○
Renal Signs	○	○	○
Ocular Signs	○	○	○
Neuro + Renal	○	○	○
Neuro + Ocular	○	○	○
Renal + Ocular	○	○	○

10. Have you seen GI stasis in the absence of other more expected clinical signs with E. cuniculi infection? *

○ Yes

○ No

11. If yes to question 10, in what percentage of these cases do you see recurrence of GI stasis?

0	1	2	3	4	5	6	7	8	9	10

0% 100%

12. If yes to question 10, do you treat these patients differently that the protocol you normally use with E. cuniculi infection?

○ Yes

○ No

13. If Yes to question 12, what other medications do you use?

14. Of the clinical signs you see in patients with ocular E. cuniculi, what is the most commonly observed presentation?

- ◯ Uveitis only
- ◯ Glaucoma only
- ◯ Cataract only
- ◯ Uveitis and cataract
- ◯ Glaucoma and cataract
- ◯ Uveitis, glaucoma and cataract

15. What kind of treatment do you most commonly use to treat these conditions?

	Medication
Uveitis only	◯
Glaucoma only	◯
Cataract only	◯
Uveitis and cataract	◯
Glaucoma and cataract	◯
Uveitis, cataract, and glaucoma	◯

16. When you see ocular signs of E. cuniculi infection, do you generally refer the patient of a veterinary ophthalmologist?

- ◯ Yes
- ◯ No
- ◯ Sometimes

17. Any other comments are welcome

18. Record your email here

This content is neither created nor endorsed by Microsoft. The data you submit will be sent to the form owner.

Microsoft Forms
